# Maintenance DFMO Increases Survival in High Risk Neuroblastoma

**DOI:** 10.1038/s41598-018-32659-w

**Published:** 2018-09-27

**Authors:** Giselle L. Saulnier Sholler, William Ferguson, Genevieve Bergendahl, Jeffrey P. Bond, Kathleen Neville, Don Eslin, Valerie Brown, William Roberts, Randal K. Wada, Javier Oesterheld, Deanna Mitchell, Jessica Foley, Nehal S. Parikh, Francis Eshun, Peter Zage, Jawhar Rawwas, Susan Sencer, Debra Pankiewicz, Monique Quinn, Maria Rich, Joseph Junewick, Jacqueline M. Kraveka

**Affiliations:** 1 Helen DeVos Children’s Hospital at Spectrum Health, Grand Rapids, USA; 2 Michigan State University College of Human Medicine, East Lansing, USA; 3 Saint Louis University School of Medicine, St. Louis, USA; 4 Arkansas Children’s Hospital, Little Rock, USA; 5 Arnold Palmer Hospital for Children, Orlando, USA; 6 Penn State Health Children’s Hospital at the Penn State Milton S. Hershey Medical Center, Hershey, USA; 7 Rady Children’s Hospital San Diego and UC San Diego School of Medicine, San Diego, USA; 8 Kapiolani Medical Center for Women and Children, Honolulu, USA; 9 Levine Children’s Hospital, Charlotte, USA; 10 Connecticut Children’s Medical Center, Hartford, USA; 11 Phoenix Children’s Hospital, Phoenix, USA; 12 Children’s Hospitals and Clinics of Minnesota, Minnesota, USA; 13 Medical University of South Carolina, Charleston, USA

**Keywords:** Paediatric cancer, Paediatric cancer

## Abstract

High risk neuroblastoma (HRNB) accounts for 15% of all pediatric cancer deaths. Despite aggressive therapy approximately half of patients will relapse, typically with only transient responses to second-line therapy. This study evaluated the ornithine decarboxylase inhibitor difluoromethylornithine (DFMO) as maintenance therapy to prevent relapse following completion of standard therapy (Stratum 1) or after salvage therapy for relapsed/refractory disease (Stratum 2). This Phase II single agent, single arm multicenter study enrolled from June 2012 to February 2016. Subjects received 2 years of oral DFMO (750 ± 250 mg/m^2^ twice daily). Event free survival (EFS) and overall survival (OS) were determined on an intention-to-treat (ITT) basis. 101 subjects enrolled on Stratum 1 and 100 were eligible for ITT analysis; two-year EFS was 84% (±4%) and OS 97% (±2%). 39 subjects enrolled on Stratum 2, with a two-year EFS of 54% (±8%) and OS 84% (±6%). DFMO was well tolerated. The median survival time is not yet defined for either stratum. DFMO maintenance therapy for HRNB in remission is safe and associated with high EFS and OS. Targeting ODC represents a novel therapeutic mechanism that may provide a new strategy for preventing relapse in children with HRNB.

## Introduction

Neuroblastoma (NB) is the most common non-CNS pediatric solid tumor, occurring in one in 7000 children. Children with low- or intermediate-risk NB have an excellent prognosis with moderate courses of chemotherapy and/or surgical resection. In contrast, children with high-risk features (approximately 50% of cases) have five year event free survival (EFS) rates of 40–60% and overall survival (OS) rates of 55–75%, despite very intensive treatment regimens that typically include five to eight cycles of chemotherapy, maximal safe surgical resection of the tumor, one or two cycles of high-dose chemotherapy with autologous stem cell support, radiation therapy, and anti-GD2 antibody plus *cis*-retinoic acid^1–6^. Even for children who successfully complete this therapy, survival is problematic: the most recent data published from the Children’s Oncology Group (COG) showed that for those who responded to induction therapy and continued to consolidation and maintenance therapy, the EFS from the start of immunotherapy was 66% ± 5% at two years^5^, but dropped to 59% ± 5% at 4 years^7^, thus demonstrating the need for further improvements in treatment. Those who relapse following front-line therapy, or who are refractory to initial therapy, often respond transiently to additional interventions but have a high rate of subsequent relapse, generally 80–90% within 2 years^8–10^. Thus, prevention of post-therapy relapse may provide an important strategy to improve survival of high risk NB patients.

Difluoromethylornithine (DFMO) is an irreversible inhibitor of ornithine decarboxylase (ODC), the rate-limiting enzyme involved with polyamine biosynthesis^11^. Elevated ODC expression and high polyamine content have been shown in NB and other tumors, and suppression of polyamine levels by DFMO reduces tumor proliferation *in vitro* and in xenograft models^12–15^. ODC inhibition by DFMO also reverses an important cancer stem cell (CSC) pathway by decreasing LIN28 and increasing Let7, and results in decreased *in vitro* neurosphere formation in neuroblastoma cell lines^16^ as well as in limiting dilution assays in xenograft models^17^. These findings provide the rationale to test DFMO as a maintenance therapy to prevent relapse in NB, at least in part by the potential to target NB stem cells.

DFMO was previously tested in combination with etoposide in a Phase I study of children with relapsed/refractory NB^18^. Subjects received DFMO for up to two years, with the addition of oral etoposide during weeks 4–15 of therapy. Responses were seen at all dose levels tested, with no apparent association between DFMO dose and response. While the median progression free survival (PFS) for all 18 evaluable subjects on this study was 80.5 days (95% CI: 62–418 days), three subjects completed 2 years of DFMO therapy and, without any additional treatment, remain alive and free of relapse over 6–8 years from completion of DFMO. This suggests that a subset of children with active NB may achieve a durable response and long- term remission from DFMO.

This phase II study was designed to evaluate the impact of DFMO on event-free and overall survival when given as a maintenance therapy to children with high risk NB who had no evidence of active disease or recurrence following completion of either standard upfront therapy, augmented treatment for refractory disease, or salvage therapy for relapsed disease, all of which are associated with a moderate to high risk of relapse.

## Materials and Methods

### Study Design, Subjects, Treatment

This was a single arm Phase II open label, single agent, multicenter clinical trial for subjects with HRNB who had completed standard therapy or therapy for refractory/relapsed disease. Subjects were enrolled onto the Beat Childhood Cancer Trial NMTRC003/003B from June 2012 to February 2016. This trial was approved by the Western Institutional Review Board as well as by local Institutional Review Boards at 22 enrolling hospitals across the United States (Saint Louis University Institutional Review Board, University of California San Diego Human Research Protections Program, Orlando Health/Orlando Regional Healthcare System Institutional Review Board, Medical University of South Carolina Institutional Review Board for Human Research, Chesapeake Research Review, Inc., Spectrum Health Institutional Review Board, Connecticut Children’s Medical Center Institutional Review Board, Children’s Mercy Hospital Pediatric Institutional Review Board, Institutional Review Board for Human Subjects for Baylor College of Medicine and Affiliated Hospitals, Vanderbilt University Human Research Protection Program, Phoenix Children’s Hospital Institutional Review Board, Seton Healthcare Institutional Review Board, Tufts University Health Sciences Institutional Review Board, University of Utah Institutional Review Board, Institutional Review Board of Children’s Hospitals and Clinics of Minnesota, Penn State Milton S. Hershey Medical Center – Penn State College of Medicine – Human Subjects Protection Office - Institutional Review Board, University of Texas Southwestern Medical Center Institutional Review Board, Johns Hopkins Medicine All Children’s Hospital Institutional Review Board, Albert Einstein College of Medicine Institutional Review Board and The Research Institute at Nationwide Children’s Hospital). Prior to study entry, written informed consent from the subject (if 18 or over), or from a parent and/or legal guardian (if under 18 y.o.) for study participation was obtained on all subjects. All methods were performed in accordance with relevant guidelines and regulations. ClinicalTrials.gov Identifiers: NCT01586260 Unique ID: NMTRC 003 Released 4/24/2012 as well as NCT02395666 Unique ID: NMTRC003B Released 3/5/2015.

Eligibility criteria included histologically confirmed diagnosis of neuroblastoma with high risk disease according to the International Neuroblastoma Risk Group Classification^19^. In addition, subjects in Stratum 1 must have completed standard high-risk NB therapy, defined as 5–6 cycles of chemotherapy based on Children’s Oncology Group (COG), Memorial Sloan Kettering Cancer Center (MSKCC), or International Society of Pediatric Oncology Europe Neuroblastoma (SIOPEN) regimens, followed by surgical resection of the primary tumor, 1–2 cycles of high-dose chemotherapy with autologous stem cell support (except for subjects treated according to MSKCC protocols, who accounted for only 4% of enrolled subjects), radiation, and anti-GD2 antibody therapy combined with retinoic acid. Criteria for enrollment on Stratum 2 was completion of any therapy for relapsed disease, or any augmented therapy for primary refractory disease (defined as any subject who received additional therapy due to a suboptimal response to standard therapy). Criteria for both strata included age at diagnosis under 21 years; adequate hematologic parameters and organ function; a disease status of at least PR (by CT or MRI) at the time of study entry and histologically negative bone marrow aspirate/biopsy. Subjects with stable residual tumor masses visible on CT/MRI were enrolled if the residual mass was either MIBG negative or MIBG positive without FDG-PET avidity, which was taken as evidence that the mass did not represent active disease and would otherwise not have received additional therapy after antibody therapy. Initiation of DFMO was required within 120 days from completion of previous therapy.

Subjects were prescribed oral DFMO continuously for twenty-seven 4-week cycles (2 years). DFMO was provided as 250 mg tablets. A dosing table was used to provide an actual prescribed dose of 750 ± 250 mg/m^2^/dose twice daily. Dosing diaries were completed for each cycle.

### End points and Assessments

The primary endpoint was EFS from first dose of DFMO; secondary objectives included OS and safety. Disease evaluations were performed every 3 months during the first year, every 6 months during the second year, then annually for 3 years after completion of DFMO. Physical examinations, adverse event assessments, and laboratory testing (including urinary catecholamines) were performed monthly while on DFMO.

### Statistical Analysis

*Survival and co-variate analysis:* Event-free and overall survival were estimated using the method of Kaplan and Meier while standard errors were estimated using Greenwood’s formula. Hypothesis testing to evaluate the effect of covariates on EFS and OS was based on the log-rank test and Monte Carlo simulation. The association of genotype with survival was tested using a two-sided stratified log-rank test for trend.

## Results

### Subject Characteristics

Consent was obtained from 111 high risk neuroblastoma subjects in Stratum 1 and 39 subjects in Stratum 2 at 21 clinical sites across the US; 101 in Stratum 1 and 39 in Stratum 2 were determined eligible for enrollment. Of the ten Stratum 1 screen failures, four had demonstrable progression of NB, five did not complete screening procedures within the mandated 120-day window following completion of antibody/*cis*-retinoic acid therapy, and one had a systemic fungal infection. Response to induction therapy was not an eligibility criterion.

Most subjects enrolled on Stratum 1 had either no evidence for persistent tumor masses, or residual tumor that was no longer MIBG avid (therefore either VGPR or PR). However, 16 of 111 screened subjects did have radiographic evidence of MIBG-positive residual disease; however, all of these underwent FDG-PET scanning that did not show increased metabolic activity in the residual masses, and thus all of these patients were enrolled. Characteristics of eligible subjects are summarized in Table 1.

**Table 1:** Subject Characteristics.

Characteristics NMTRC003/003B		Stratum 1 (n = 101)	Stratum 2 (n = 39)
Mean Age at diagnosis		3·5 years	3·2 years
Sex	Male	57 (56%)	28 (72%)
	Female	44 (44%)	11 (28%)
Ethnicity	White	72	32
	Black or African American	7	3
	American Indian/	2	1
	Alaska Native		
	Hispanic	10	1
	Asian	0	0
	More than one	3	0
	Unknown	7	2
Stage at Diagnosis		2: 2 (2%) (All *MYCN*++)	0
		3: 6 (6%)	0
		4: 93 (92%)	39 (100%)
*MYCN*		Amplified: 47 (48.4%)	7 (21.2%)
		Non-Amplified:50 (51.5%)	26 (78.8%)
		Unknown: 4	6
Histology		Unfavorable: 48 (90.6%)	2 (22.2%)
		Favorable: 5 (9.4%)	7 (77.8%)
		Unknown: 48	30
Ploidy		>1: 17 (50%)	2 (28.6%)
		=1: 17 (50%)	5 (71.4%)
		Unknown: 67	32
Median Time from diagnosis to DFMO		1·3 years	3·4 years
Response to induction therapy		CR: 42 (48.8%)	
		VGPR: 22 (25.6%)	
		PR: 20 (23.2%)	
		SD: 2 (2.3%)	
		Unknown: 15	
Number of ASCTs		00:04	
		0.104166667	
		02:07	

**Figure 1:**
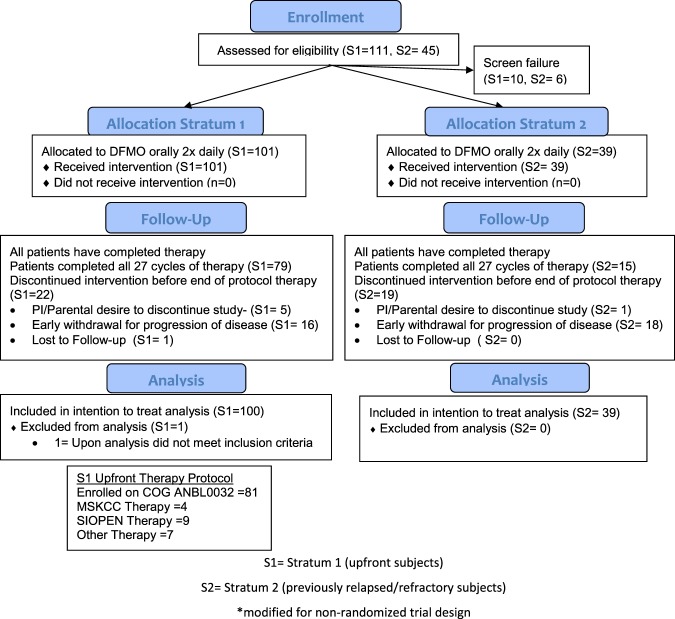
NMTRC003 CONSORT Flow Diagram.

All 140 eligible subjects received DFMO and were eligible for safety analysis. After central review one stratum 1 subject did not meet inclusion criteria due to starting DFMO > 120 days after the completion of standard therapy; this subject was excluded from the ITT analysis but did complete DFMO therapy with relapse. Thus, 139 subjects were eligible for the intention to treat (ITT) analysis (Fig. 1).

#### Stratum 1

Subjects had received various standard upfront treatment regimens, including those enrolled on or treated as per COG protocols (81%), MSKCC (4%), SIOPEN (9%), and other (6%). Within the ITT population, 81 subjects had previously enrolled and completed antibody therapy on the post-randomization extension of COG ANBL0032. Patients without active disease or recurrence on post-antibody scans were eligible. As previously noted, sixteen subjects had residual stable MIBG-avid tumor at the end of antibody therapy and were further evaluated by PET; all were PET negative and therefore no subjects were excluded because of the presence of stable residual disease.

High risk features of our subject population were similar to those previously reported for HRNB population studies with regards to stage, *MYCN*, histology, ploidy and induction response^5,20^. All subjects enrolled in the ITT populations were compliant with study visits and evaluations, with 98% of subjects receiving >80% of DFMO doses over the two years of treatment.

#### Stratum 2

Subjects had received various upfront and relapse therapies, with 21 treated for refractory disease and 17 treated for relapsed disease. Within the relapse group, subjects had received a mean of 3.3 prior additional therapeutic regimens and experienced a mean of 1.7 prior complete remissions. The overall mean time from diagnosis to enrollment was 3.4 years; 3.9 years for relapsed subjects and 2.9 years for subjects with primary refractory disease.

### Outcome

#### Stratum 1

**Figure 2:**
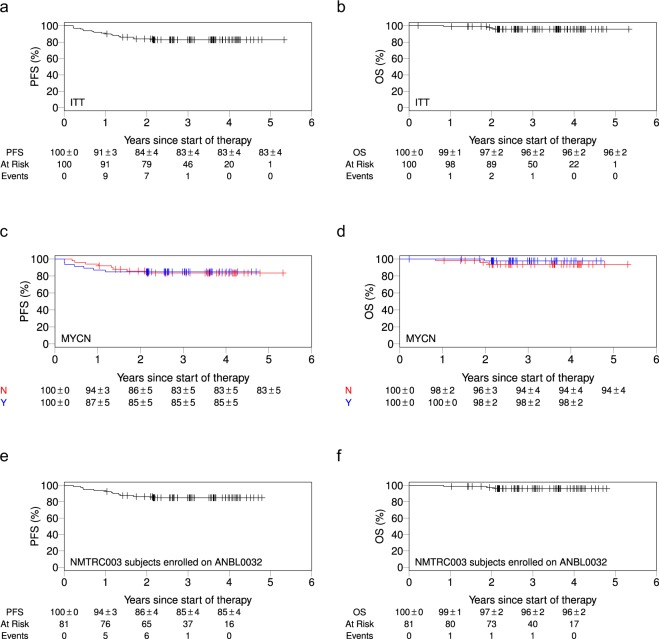
Event free survival and overall survival for the Stratum 1 intention to treat (ITT) population. (**a**) Event free survival and (**b**) overall survival for all subjects. (**c**) Event free survival and (**d**) overall survival for *MYCN* amplified versus non-amplified subjects. (**e**) Event free survival and (**f**) overall survival for all subjects previously enrolled on ANBL0032.

Among all subjects in Stratum 1 who received DFMO, the two-year EFS was 84% ± 4% and OS was 97% ± 2% (Fig. 2a,b). The median follow-up time is 3.5 years (range 2.1–5.8 years). EFS and OS were similarly high regardless of high risk features, including *MYCN* status (Table 1, Fig. 2c,d). The subset of subjects that received DFMO following completion of the COG ch14:18 antibody clinical trial (ANBL0032, n = 81) had a 2-year EFS of 86% ± 4%, (Fig. 2e) and OS of 97% ± 2% (Fig. 2f).

#### Stratum 2

**Figure 3:**
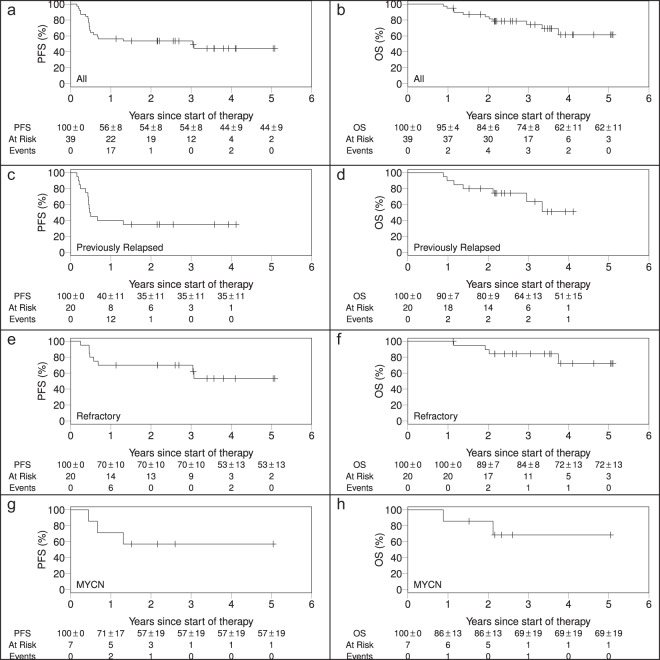
(**a**) Event free survival and (**b**) overall survival for the Stratum 2 intention to treat (ITT) population. (**c**) Event free survival aRnd (**d**) overall survival for previously relapsed subjects in Stratum 2. (**e**) Event free survival and (**f**) overall survival for previously refractory subjects. (**g**) Event free survival and (**h**) overall survival for MYCN amplified versus non-amplified subjects.

Among all Stratum 2 subjects, the two-year EFS was 51% ± 8% and OS was 84% ± 6% (Fig. 3a,b) with a median follow-up time of 3.7 years (range 2.1–5.8 years). Previously relapsed subjects (N = 18) had an EFS of 35% ± 11% and OS of 80% ± 9% at two years (Fig. 3c/d). Subjects with primary refractory disease (N = 21) had an EFS of 68% ± 11% and OS of 89% ± 7% at two years (Fig. 3e/f).

### ODC SNP Analysis

Single nucleotide polymorphisms (SNPs) in the ODC gene have been associated with risk of specific cancers^21,22^. The minor A allele at rs2302615 in the ODC gene was found to be a risk allele for survival in patients with prior colorectal cancer^23^, but a protective allele in patients with NB^24^. The results from our Phase I study suggested that genetic variability affecting ODC expression, specifically the rs2302616 SNP, was associated with increased polyamines, enhanced susceptibility to the ODC inhibitor DFMO and subsequent increased responsiveness to DFMO containing therapies in patients with NB^18^ (although the association of genotype with event-free survival did not reach statistical significance). However, in this study we found no significant association between rs2302616 genotype and EFS or OS (p = 0.96). (Table 2).

**Table 2:** Tests of the association of survival with ODC1 single nucleotide polymorphism rs2302616 genotype.

Factor Levels	p-value	p-value
	EFS	OS
GG, GT, TT	0.96	0.38
GG or GT, TT	0.58	0.29
GG, GT or TT	0.67	0.63

### Adverse Events

DFMO was well tolerated without any serious adverse events (Table 3). 67% of subjects reported no treatment-related adverse events. The most common reported toxicity was Grade 2-3 transaminitis (<10 X ULN). Of note, this toxicity did not necessitate the holding of DFMO treatment and resolved with continued treatment. 76% of subjects had pre-existing hearing loss at study entry, and during this study 5 (4%) subjects had an increase in hearing loss that required temporarily holding DFMO. Hearing loss with DFMO is reversible and returned to baseline levels in all subjects, and all were able to complete DFMO treatment without further hearing loss. One subject experienced Grade 4 hypoglycemia during an episode of viral gastritis (vomiting and inability to tolerate feeds) while on DFMO, which therefore was considered possibly treatment-related. The patient was restarted on DFMO without further episodes of hypoglycemia.

**Table 3:** Adverse events attributed (possibly, probably, or definitely) to DFMO.

n = 140	Grade 2	Grade 3	Grade 4	Grade 5
**Hematologic Toxic Effects**				
Anemia	4 (3%)	2 (1%)	~	~
Neutrophil count decrease	7 (5%)	4 (4%)	~	~
Platelet count decrease	2 (1%)	~	~	~
White blood cell decreased	3 (2%)	~	~	~
**Non-hematologic Toxic Effects**				
Abdominal Pain	1 (<1%)	~	~	~
Agitation	1 (<1%)	~	~	~
Alopecia	2 (1%)	~	~	~
ALT elevation	7 (5%)	5 (4%)	~	~
AST elevation	5 (4%)	4 (4%)	~	~
Alkaline phosphatase elevation	1 (<1%)	~	~	~
Anorexia	1 (<1%)	~	~	~
Diarrhea	6 (4%)	1 (<1%)	~	~
Fever	4 (3%)	~	~	~
Hearing Loss	1 (<1%)	5 (4%)	~	~
Hypoglycemia	~	~	1 (<1%)	~
Hypokalemia	~	2 (1%)	~	~
Infection, Other	3 (2%)	~	~	~
Infection, middle ear	6 (4%)	~	~	~
INR Elevated	1 (<1%)	~	~	~
Insomnia	1 (<1%)	~	~	~
Pain	2 (1%)	~	~	~
Post Nasal Drip	1 (<1%)	~	~	~
Rash	3 (2%)	~	~	~
Vomiting	~	1 (<1%)	~	~
Weight Gain	1 (<1%)	~	~	~

ALT = alanine aminotransferase AST = aspartate aminotransferase.

## Discussion

The primary objective of NMTRC003 was to evaluate the EFS of children with HRNB who received DFMO as maintenance therapy either after completion of standard therapy for high risk NB or after therapy for relapsed/refractory disease. Subjects received 750 ± 250 mg/m^2^ twice daily of DFMO for 2 years. Those treated after completion of standard therapy started DFMO at a median of 1.2 months from the last dose of retinoic acid and demonstrated a two-year EFS of 84% and OS of 97% with minimal and easily managed toxicity. Those receiving DFMO after therapy for relapse/refractory disease had a two-year EFS of 51% and OS of 84%.

The dose of DFMO in this study was chosen based on results from our previous phase I clinical trial for HRNB subjects^18^ in which responses were seen at all dose levels and pharmacokinetic studies demonstrated an overall maximum serum DFMO concentration of 14.2 ± 7.9 mcg/mL in subjects receiving 750 mg/m^2^. Previous studies in adults have shown that DFMO at this concentration is effective and has achieved desired biological activity as demonstrated by a decrease in urinary polyamines and ODC activity, and has been described as suitable target concentration for metronomic therapy^18,25,26^.

This single arm Phase II study was initiated following completion of a Phase I trial in relapsed NB. Despite several limitations, contemporaneous controls have been successfully used to evaluate the results of previous high risk neuroblastoma clinical trials^25,26^. Therefore to evaluate comparable subjects, we used a subset of subjects enrolled (81% of those on Stratum 1) who immediately prior to enrollment on this study received anti-GD2 immunotherapy while enrolled on the single-arm non-randomized extension of COG ANBL0032. Covariate analysis of population risk variables were matched including time from diagnosis to enrollment on study, initial disease stage, *MYCN* status, age at diagnosis, and response to induction therapy^5^. The median time from start of antibody to start of DFMO was 7.2 months. After statistical correction for this “run in” period, the proportion of ANBL0032 patients remaining event-free for an additional 2 years (the duration of DFMO therapy) is conservatively estimated at 75%, and EFS continues to decrease between 2 and 4 years post therapy. In contrast, the observed two-year EFS for subjects in Stratum 1 who received DFMO after ANBL0032 therapy was 86% ± 4%, and survival curves are stable to 4 years. This suggests there is a clinically significant benefit to maintenance DFMO, justifying further study in a confirmatory Phase II study.

Given that the entire cohort includes subjects who had received various standard-of-care therapies prior to starting DFMO, it is important to review the outcomes from other groups. While numbers are small, analysis of the subject cohorts that received non-COG frontline therapies also suggests benefit for DFMO maintenance therapy. A European study^3^ reports that high risk NB patients who were treated with a similar overall therapy strategy (including anti-GD2 antibody) had 3 year EFS of 46.5% ± 4.1% and OS of 86.5% ± 3.9%^3^; analysis of their survival curves to correct for a lead-in period suggests that those who were event free at the completion of antibody therapy experienced a subsequent 2-year EFS of <60%. Similarly, MSKCC reported on the survival of Stage IV patients who were further stratified into “high-risk” and “ultra-high-risk” groups. While analysis of the high risk cohort (excluding ultra-high risk patients) showed EFS of 69% and OS of 78% at 5 years post start of antibody (with an estimated EFS of 84% at two years following completion of antibody therapy), comparable analysis for the ultra-high-risk group were EFS of 44% and OS of 78% at five years post start of antibody, and an EFS of approximately 54% at two years following completion of antibody therapy^6^.

Treatment for relapsed/refractory NB involves many therapeutic approaches, often involving Phase I and II chemotherapy trials. These generally have, at best, conferred modest response rates with high rates of subsequent relapse. A recent review of studies determined that historical rates of progression free survival (PFS) and OS for relapse therapies at 1 and 4 years were 21% ± 2% and 6% ± 1%, respectively, and the OS rates were 57% ± 3% and 20% ± 2%, respectively. Outcomes were worse for those with tumors having MYCN amplification: 1- and 4-year PFS of 13% ± 6% and 0%, respectively, and OS of 30% ± 8% and 0%, respectively^10^. In contrast, relapsed/refractory patients with MYCN-amplified tumors in our study had 1- and 4-year PFS of 71% ± 17% and 57% ± 19%, respectively, and OS of 86% ± 13% and 69% ± 19%, respectively. This suggests that DFMO may benefit these especially challenging patients. Of note, a recent Phase I study at MSKCC evaluating administration of an anti-tumor vaccine following standard therapy suggested that alternative maintenance therapy to prevent relapse in high-risk patients may be beneficial^27^. Thus, our findings add support to the concept that aggressive salvage programs for relapsed/refractory HRNB are warranted as disease cure may still be possible.

Our study indicates that there may be a long-term benefit to DFMO maintenance therapy even in those who have relapsed or responded poorly to standard induction therapy—groups that historically have had poor long-term survival. This effect appears to be greater for subjects with primary refractory disease than those who initially responded to treatment and then experienced a relapse, a difference that is currently being studied further in a follow up clinical trial with each cohort being studied independently. In addition, these groups in particular may merit evaluation of early combination of DFMO with standard chemotherapy in an effort to produce a sustainable remission and thus further improve survival rates.

Rather than exerting a direct cytotoxic effect on tumor cells, DFMO exhibits a unique mechanism of action in that it inhibits ODC activity and reverses the effects of increased polyamine levels^28,29^. *MYCN*, which is amplified in approximately one-third of neuroblastomas and associated with high-risk behavior, is a transcription factor that regulates gene expression of ODC. Thus, it has been suggested that targeting ODC with DFMO would have greater effect on *MYCN* amplified tumors^28,29^. However, subjects on this study showed improvement in EFS and OS regardless of *MYCN* status (Fig. 2c/d, Table 1), indicating that while DFMO’s targeting of ODC may result in a relatively greater benefit for the *MYCN* amplified group, subjects without *MYCN* amplification also benefit. This may be as a result of DFMO’s decrease of LIN28 which may affect the expression of multiple additional oncogenes through correction of the LIN28/Let7 axis derangement^30^.

Finally, although current treatment strategies for high-risk NB have resulted in improvement in response rates, long-term EFS and OS remain disappointing and many survivors have profound cumulative toxicities, including cardiotoxicity, ototoxicity, hypothyroidism, second malignancies and post-transplant complications that would limit further intensification of conventional therapy^31,32^. The schedule of maintenance DFMO utilized in the current trial appears to improve survival while demonstrating manageable toxicity. DFMO has been used for >30 years by the World Health Organization for African sleeping sickness with a well-established safety profile. A prior phase I study in adults given doses of 3750 mg/m^2^/day—five times higher than the doses used in the current study—demonstrated no clinically significant renal, hepatic, auditory or hematologic toxicities^33^.

In summary, by inhibiting ODC and decreasing intracellular polyamine levels, DFMO targets a novel pathway and therefore is likely to provide a novel therapeutic strategy for maintaining long-term remission in children with high-risk neuroblastoma. DFMO given to children with high risk NB following completion of either standard multimodal therapy or salvage therapy for relapsed/refractory disease was both well tolerated and appeared to improve the event free and overall survival rates when compared to historical controls. A confirmatory Phase II and a randomized clinical trial utilizing DFMO during immunotherapy are both ongoing.

## Data Availability

The datasets generated during the current study are available in the clinicaltrials.gov repository, https://clinicaltrials.gov/ct2/show/NCT02395666.
